# Improving EMS access: Strategic helicopter infrastructure and 3D virtual training for enhanced ground-air ambulance coordination

**DOI:** 10.1016/j.isci.2025.114569

**Published:** 2026-01-07

**Authors:** Soyoung Jung, Xiao Qin, Sooyeon Lim

**Affiliations:** 1 School of Smart Safety System, Dongyang University, 2741 Pyeonghwaro, Dongducheon, Gyeonggido 11307, South Korea; 2 Department of Civil and Environmental Engineering, University of Wisconsin-Milwaukee, NWQ 4414, P.O. Box 784, Milwaukee, WI 53201, USA; 3 Department of Fine Arts, Kyungpook National University, 80 Daehakro, Daegu 41566, South Korea

**Keywords:** Data science, earth science, health science

## Abstract

This study aimed to develop a methodology to strengthen coordination between ground ambulances and helicopter emergency medical services (EMS) for rapid patient transport. Using data on helicopter EMS infrastructure, crash locations, and prehospital EMS times, safety assessments and geographic information system (GIS)-based network analyses were conducted to identify service gaps and prioritize infrastructure expansion. Results showed that EMS stations and heliports near high-fatality crash locations were insufficient to meet prehospital EMS time thresholds. To address this, a three-dimensional virtual space platform combining GIS-based spatial modeling with real-time visualization was developed for training ground and air ambulance operators. This platform realistically simulates patient transport scenarios, enhancing decision-making, and collaboration. The findings provide a data-driven framework for optimizing helicopter EMS infrastructure and developing practical training tools, offering policymakers quantitative evidence to improve EMS accessibility and promote effective strategies for timely patient transport.

## Highlights

• Framework to assess helicopter EMS investment and design a 3D virtual EMS access training
• Data-driven safety and GIS analysis for strategic helicopter EMS systems
• 3D virtual EMS training platform integrating GIS space models and real-time visualization
• Research guiding policymakers to balance EMS infrastructure and transport training

## Graphical abstract

**Figure:**
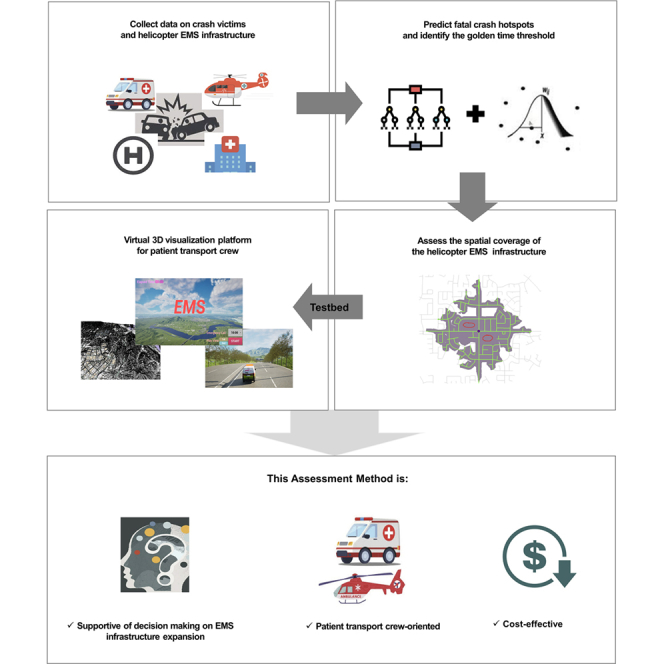

## Introduction

Timely emergency medical services (EMS) are critical for providing patients with essential care, reducing transport time and improving survival rates, particularly for rapid access to rural areas as evidenced by several studies.^1^^,^^2^^,^^3^^,^^4^ However, inadequate helicopter EMS (HEMS) infrastructure and insufficient training for helicopter transport undermine the speed of EMS access to patients compared to their urban counterparts.

In South Korea (hereafter referred to as Korea), local governments are responsible for constructing and managing helicopter landing sites in their jurisdictions. They face challenges such as budgetary constraints and residents’ complaints about helicopter noise. Additionally, air ambulances are required to land only at official heliports (referred to as predesignated heliports in Korea), and night operations for air ambulances are prohibited.^5^

The limited HEMS infrastructure and operations also create significant spatial disparities in EMS access. According to the Korean Statistical Information Service, the fatality rate per crash on Korean urban roads from 2018 to 2022 was about one-third of that on rural roads. Currently, Korea is divided into nine provinces, and HEMS infrastructure is unevenly distributed. Nearly 35% of all EMS hospitals are concentrated in the capital province due to its high population density. The average prehospital EMS times—defined as the time from when an emergency call is received to a patient’s arrival at a definitive hospital^6^ are 36 min in urban areas and 42 min in rural areas.^7^ This indicates a significant gap in the timely care of trauma injuries.^8^ Consequently, the coefficient of variation in the death rate of patients transported from road crashes is approximately 30% across the nine provinces.

To deal with these issues of EMS accessibility, the Korea Ministry of Health and Welfare (KMHW) announced an emergency medical master plan in 2023.^9^ The KMHW plan focuses on expanding EMS infrastructure, particularly for transporting severely injured patients. It includes increasing the number of air ambulances and designating additional EMS hospitals (referred to as EMS centers hereafter). Since 2022, the Korea National Fire Agency (KNFA) has also outlined major work plans, including increasing the number of EMS stations in rural areas and equipping EMS helicopters with specialized medical doctors on board.^10^

However, there have been no quantitative studies that comprehensively assess the state of HEMS-related infrastructure, including EMS stations, EMS centers, EMS helicopters as air ambulances, and their landing spots. A Korean analysis report indicated that rescues from severe road crashes are the most economically effective among all diseases or incidents.^11^ Despite this, there have been few data-driven studies on road crashes in Korea. While some studies in other countries have examined EMS infrastructure coverage using road crash data,^12^^,^^13^ they have not comprehensively considered the distribution of HEMS-related infrastructure, such as heliports. Moreover, there is no guidance on how to improve ground and air ambulance coordination to enhance EMS accessibility especially around crash sites with a high potential for severe injuries.

Strategic improvements to HEMS infrastructure and management are crucial for achieving equitable EMS accessibility. A virtual 3D visualization platform for training HEMS operators is an integral component of this strategy. Such a platform can replicate real-world environments with high fidelity, providing HEMS operators with a realistic experience in navigating patient transport routes. It allows for training that can be accessed from anywhere and at any time, offering flexibility and convenience. Additionally, virtual training can significantly reduce fuel and maintenance costs compared to traditional training with real helicopters and equipment. Effective patient transport from crash sites to definitive hospitals requires seamless coordination between EMS vehicle drivers and helicopter pilots. A virtual 3D platform can facilitate team-based training exercises, enhancing communication, and collaboration skills among team members.

To date, multiple studies have addressed the necessity of HEMS operations. A previous study by Shahriari et al.^14^ verified that HEMS improves the patient transport system from the car crash location to a definitive hospital comparing to no system applied.

When focusing on road crash victim transport, however, few studies have comprehensively examined HEMS infrastructure accessibility. Some studies measured road crash data based EMS infrastructure coverage,^12^^,^^13^ these study did not comprehensively consider helicopter related infrastructure such as heliports. Only a Korean study^15^ considered the distributions of heliports and sub-heliports to assess road crash victim transport to an EMS center by HEMS access. Most studies have focused on the demand in ground ambulance response to road crash victims.^16^^,^^17^^,^^18^

In the meantime, Geographical Information System (GIS) based analysis has been widely used for measuring EMS access to more broad targets. The GIS techniques used to measure EMS accessibility included straight line buffer based spatial coverage,^19^^,^^20^ network-based catchment area or temporal distance,^21^^,^^22^^,^^23^^,^^24^ and decaying density functions modeling the EMS accessibility with distance.^25^ Specifically considering the network based catchment area or temporal distance measurement approach, one study employed service area technique to quantify EMS infrastructure access to natural disaster scenarios. In the process of EMS infrastructure coverage measurement, the study conducted model building to identify high-priority locations for EMS access.^26^

Furthermore, a three-dimensional (3D) virtual environment has been developed to realistically simulate HEMS accessibility for immersive patient transport training of ground and air ambulance personnel, based on real-time data collection, processing, analysis, and visualization. In light of the rapid advancement of digital twin technology, GIS data such as geographic locations, terrain, and buildings have been extensively utilized to create precise virtual replicas of physical realities. The world’s first digital twin introduced in smart city infrastructure, “Virtual Singapore”, has significantly enhanced safety levels related to urban planning and engineering in Singapore.^27^

Due to its unparalleled realism of digital twin technology, the application of digital twin technology in road modeling and 3D traffic simulation has gained strong traction. A study by Amara et al.^28^ functionally modeled road sections and intersections based on GIS data and implemented a simulation that mimics real driver/vehicle interaction through a 3D rendering engine. Kong et al.^29^ proposed an approach to animatedly visualize spatial-temporal data using attribute-pluggable models and a modular web-based GIS.

From the research background and literature review outlined previously, our study filled the gap by comprehensively assessing the accessibility of HEMS infrastructure (including EMS vehicle, centers, helicopters, and their landing spots) to reduce road crash involved fatalities and by developing a 3D virtual platform of training exclusively for EMS vehicle and helicopter operators.

Accordingly, this research aimed to develop a method for the sustainable expansion of HEMS infrastructure and to create a virtual 3D visualization platform for training in HEMS access. This study focused on the coordination between ground EMS vehicles and air ambulances in transporting road crash victims from the crash site to a definitive hospital. Essential infrastructure for this process includes EMS stations that operate EMS vehicles, high-level hospitals (EMS centers) that provide emergency medical treatment, EMS helicopters, and heliports, collectively referred to as HEMS infrastructure.

The specific objectives in this study are to: (1) identify road crash locations that require high-priority HEMS infrastructure support; (2) quantify the spatial coverage of existing HEMS infrastructure; (3) recommend the extent of additional HEMS infrastructure needed; and (4) develop a 3D visualization platform for training in ground and air ambulance coordination for HEMS access.

## Results

The methodology for assessing prehospital HEMS accessibility and developing a corresponding 3D visualization platform consisted of the following steps:

*Step 1***:** The medical records of traffic crash victims used in this study did not contain information on HEMS infrastructure located near the crash locations. Therefore, publicly available datasets (EMS stations, hospitals, helicopter bases, predesignated heliports, and school playgrounds) were integrated with the crash location information included in the medical records. By linking the nearest HEMS infrastructure to each crash location,^30^ an expanded crash victim record dataset was constructed.
*Step 2***:** A Random Forest (RF) algorithm was applied to the expanded dataset to identify variables with strong influence on injury severity (medical treatment outcomes). The selected variables were used as predictors in a geographically weighted binary logistic (GWBL) regression model to estimate the probability of injury severity. Model performance was compared with that of a standard binary logistic (BL) regression model using model performance criteria, which confirmed the superior predictive capability of the GWBL model.
*Step 3***:** Specific response time and transport time variables identified as statistically significant predictors in the GWBL regression model were adopted as thresholds for prehospital EMS time. Based on these time thresholds, service areas were generated to assess prehospital HEMS accessibility around target locations, defined as crash sites with a high predicted probability of severe injury.
*Step 4*: The HEMS access simulation platform integrated GIS-based data with game engine to create a high-fidelity virtual environment for realistic emergency response training. The system supports selectable first- and third-person viewpoints and integrates dynamic environmental factors, including real-time sun positioning, to facilitate effective and immersive coordinated training between ground EMS vehicles and helicopters.

Additional methodological details are provided in the STAR Methods section.

### Road crash victims and HEMS infrastructure in the study area

Choongbuk (CB) province is located in central Korea and has the highest proportion of rural areas among the country’s nine provinces in Korea, with 73% of its land classified as rural.^31^ CB province also has the longest average prehospital EMS time (42 min).^7^ The current study utilized data on medical treatments for road crash victims transported to Konkuk University Hospital in CB province, from 2011 to 2016. All variables considered in the road crash victim dataset are listed in Table 1. Note that each attribute of the variables in Table 1 was converted into an indicator variable.

**Table 1:** Variable statistics

Characteristic	Variable	Attribute	Sample proportion (%)
Dependent	Medical treatment outcome	Survival	94.4
		Fatality	5.6
EMS time periods	EMS vehicle response time aggregated by the on-scene time	Less than 6 min	7.9
		6 to 10 min	35.2
		11 to 15 min	22.4
		16 to 20 min	19
		21 to 25 min	9.7
		More than 25 min	5.8
	EMS vehicle transport time	Less than 11 min	8.1
		11 to 15 min	21.8
		16 to 20 min	14.8
		21 to 25 min	12
		26 to 30 min	12.9
		More than 30 min	30.5
Spatiotemporal condition	Hour of the day	20:00 to 05:59	27.1
		6:00 to 8:59	18.1
		9:00 to 13:59	23.8
		14:00 to 16:59	17.6
		17:00 to 19:59	13.4
	Season	Spring (March to May)	28.5
		Summer (June to August)	27.6
		Autumn (September to November)	23.6
		Winter (December to February)	20.3
	Sunlight	Daytime	66.5
		Night	33.5
	Weather	Clear	72.4
		Cloudy	6.3
		Rain/Snow/Fog	21.3
	Roadway	Expressway	17.6
		National highway	13.4
		Rural principal road	13.6
		Urban road	55.4
Collision	Collision type	Single vehicle involved	12.5
		Head-on	25.9
		Angle	47.9
		Rear-end	13.7
	Primary cause	Driving under alcohol effect	11.6
		Drowsy driving	6.2
		Centerline violation	6.3
		Signal/speed/parking violations	12.4
		Careless driving	53.2
		Vehicle defects	3.3
		Roadway conditions	7
Vehicle	Victim’s vehicle type	Passenger car	52.6
		SUV/Van	30.6
		Truck	16.8
	Counterpart vehicle	Passenger car	29.8
		SUV/Van	13
		Truck	13.6
		Trailer/Bus	13.6
		Fixed objects	18.5
		Rollover	11.5
	Front airbag	Deployed	19.4
		Not deployed	80.6
	Side airbag	Deployed	5.3
		Not deployed	94.7
Victim	Age	Younger than 25 years old	16
		25 to 64 years old	71.1
		Older than 64 years old	12.9
	Gender	Male	59.3
		Female	40.7
	Seatbelt	Worn	70
		Not worn	30
	Position	Driver Front passenger	56.7 21.5
		Back	21.8

**Figure 1:**
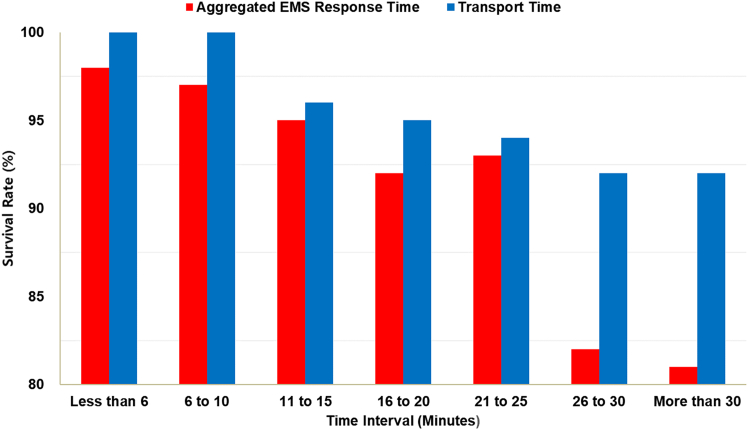
Survival rates by EMS time intervals

Based on the dependent variable (medical treatment outcome) listed in Table 1, survival rates across prehospital EMS time intervals are presented in Figure 1. The survival rate was calculated as the number of survival cases divided by the total number of cases within each time interval.

Figure 1 illustrates that survival rates decrease as both aggregated EMS response time and transport time increase, except for the 21 to 25-min interval. In this interval, 49 cases out of 55 cases had injury severity scores of less than 15, indicating that the patients were not severely injured at the time of the crash. This may explain why the survival rate trend was atypical for the aggregated EMS response time interval of 21–25 min.

**Figure 2:**
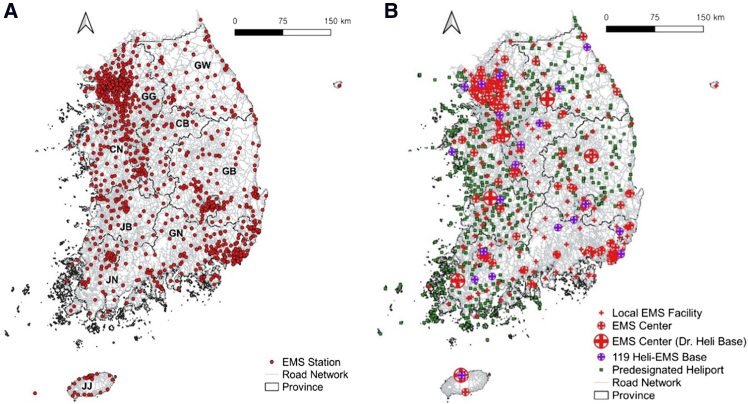
The distribution of existing HEMS infrastructure in Korea (A) EMS stations. (B) EMS centers, helicopter bases and heliports.

This study also incorporated spatial data on HEMS infrastructure for GIS-based service area generation. Figure 2 illustrates the locations of EMS stations, EMS centers serving as definitive hospital candidates, helicopter bases, and predesignated heliports available for HEMS operations across Korea.

In addition, Table 2 presents technical specifications of helicopters currently available for HEMS operations in Korea.

**Table 2:** Technical information of EMS helicopter

Affiliation	Base city (Province)	Manufacturer	Model	Max./Avg. speed (km/h)	Service range (km)	Night operation
KMHW	Suwon (GG)	Leonardo	AW169	297/268	70 to 250	No
	Incheon (GG)					
	Mokpo (JN)					
	Wonju (GW)	-	AW109	311/285	–	
	Andong (GB)					
	Cheonan (CN)					
	Iksan (JB)					
	Jeju (JJ)	Korea Aerospace Industry (KAI)	Light civil helicopter	265/265	–	
KNFA	Namyangju (GG)	Airbus	H225	324/262	250 to 400	Yes
	Yangyang (GW)	Leonardo	AW139	310/277	–	–
	Hweongsung (GW)					
	Incheon (GG)					
	Yongin (GG)					
	Taean (CN)					
	Busan (GN)					
	Youngam (JN)					
	Daegu (GB)	-	AW169	297/268	–	–
	Seoul (GG)		AW189	313/287		
	Choongju (CB)		H225	324/262		
	Daegu (GB)				-	-
	Cheongju (CB)	Kawasaki	BK117C	276/240		
	Daejeon (CN)					
	Wanju (JB)		BK117B2	278/267		
	Gwangju (JN)				-	-
	Ulsan (GN)	KumAPE	KA-32T	230/200		
	Hapcheon (GN)	KAI	KUH-1EM	290/278		
	Hwasoon (JN)					
	Jeju (JJ)					

### RF based GWBL model

**Figure 3:**
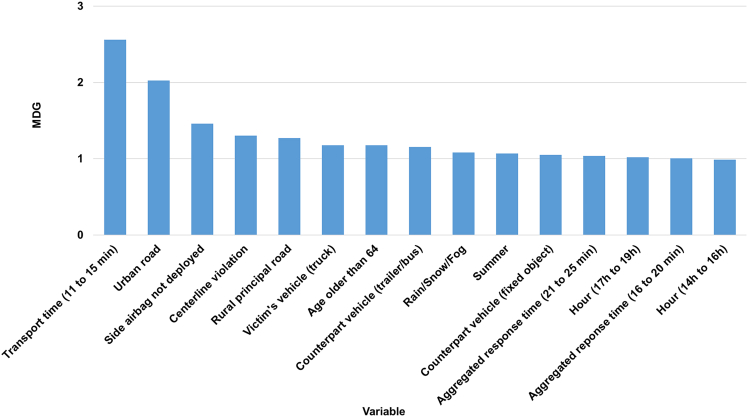
Key variable importance ranking from RF

The RF algorithm provided MDG based variable importance rankings for all independent variables. The resulting ranking for the 15 key independent variables is shown in Figure 3.

Using the key variables identified by the RF algorithm, a GWBL regression model was developed. A Gaussian adaptive kernel function was employed to generate spatial weights for the GWBL model, and the model results are presented in Table 3.

**Table 3:** Multiple regression model

Geographically weighted binary logistic								Standard binary logistic		
Parameter	Min. β	Q1. β	Q2. β	Q3. β	Max. β	Max.|z|	Mean β	β	S.E.	*p* value
Intercept	−3.93	−3.57	−3.45	−3.31	−3.19	9.91	−3.46	−3.73	0.37	0.00
11-15 min transport time	1.87	1.92	1.97	2.04	2.23	5.19	1.99	2.22	0.43	0.00
16-20 min aggregated EMS response time	0.45	0.62	0.81	0.98	1.56	3.78	0.84	1.23	0.40	0.00
Centerline violation	1.46	1.50	1.52	1.53	1.56	2.45	1.51	1.54	0.63	0.01
Urban road	−1.82	−1.67	−1.50	−1.32	−0.84	3.35	−1.47	−0.97	0.42	0.02
Deviance (intercept/fitted)	246.25/195.29							246.25/204.46		
AIC	210.09							214.57		
Pseudo R^2^	0.21							0.17		

According to Table 3, the GWBL model outperformed the standard binary logistic model in overall goodness-of-fit, with lower deviance and AIC values and a higher pseudo R^2^. The GWBL model also captures spatial variation, presenting coefficient estimates by mean, minimum, lower quartile (Q1), median (Q2), upper quartile (Q3), and maximum values, whereas the standard model does not account for spatial effects.^16^ For all four independent variables, the maximum absolute z values in the GWBL model exceeded 1.96, indicating statistical significance at the 95% confidence level.

Two prehospital EMS time variables in the GWBL model—11 to 15 min patient transport time and 16 to 20 min aggregated EMS response time—were associated with higher probabilities of crash fatalities. This suggests that crash victims are more likely to survive if initial medical care is provided within 15 min of the crash and they are transported to a hospital within 10 min. As shown in Figure 1, survival rates generally decline as EMS response and transport times increase. Therefore, 15 and 10 min were set as the respective thresholds for aggregated EMS response time and patient transport time.

**Figure 4:**
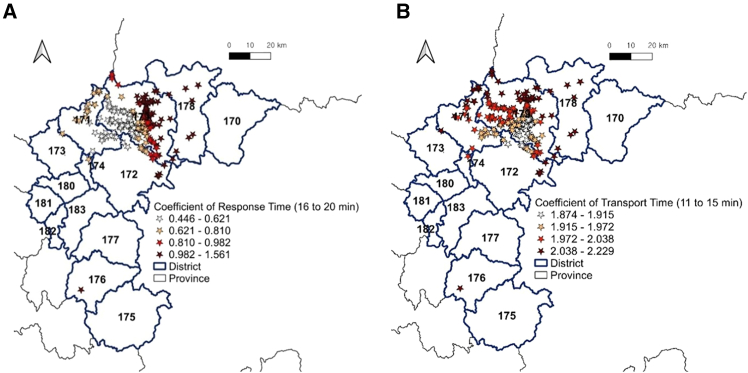
Distribution of significant coefficients for the prehospital EMS time variables in CB (A) Coefficients of 16 to 20-min aggregated EMS response time. (B) Coefficients of 11 to 15-min transport time.

Figure 4 shows the spatial distribution of the coefficient estimates for these two time variables, as reported in Table 3. Only locations with coefficients significant at the 95% confidence level are displayed.

The CB province of Korea shown in Figure 4A contains 14 administrative districts, which are three cities (particularly, the City of Cheongju including four sub-districts) and eight counties. Figures 4A and 4B exhibit the fatality-prone locations that are significantly affected by a 16 to 20-min aggregated EMS response and an 11 to 15-min patient transport time, respectively. The coefficient estimates of the two time-related variables were divided into four classes using their minimum, Q1, Q2, Q3, and maximum values.

In this study, the coefficient estimates that are greater than their Q3 values were considered to have “locally strong impacts” on crash fatality occurrences. Dark red stars in Figure 4 denote the fatality-prone locations that are strongly impacted by EMS time-related variables. In Figure 4A and 68 fatality-prone locations strongly affected by a 16 to 20-min aggregated EMS response time were distributed in the same areas, except for district No. 171 (Umseong County). In Figure 4B, sixty nine fatality-prone locations strongly affected by an 11 to 15-min patient transport time were concentrated in the northeastern and southwestern areas (districts No. 171, 172, 176, 178, and 179) of the CB province.

### GIS-based spatial network analysis

**Figure 5:**
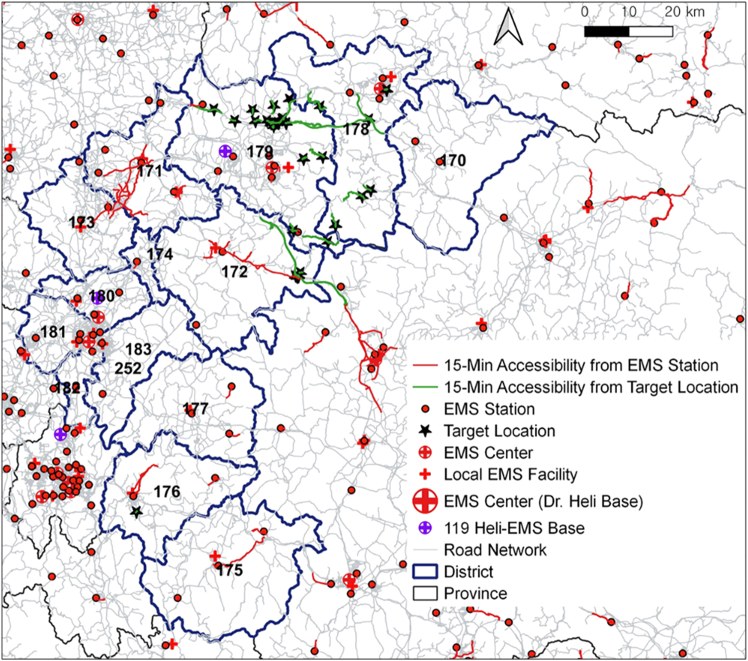
EMS vehicle accessibility originating from EMS station or target location

The research can be used to guide decision-making on where to prioritize the expansion of HEMS infrastructure to mitigate crash consequences. Locations with high fatality rates that are significantly affected by response times ranging from 16 to 20 min and transport times from 11 to 15 min were identified. Overlapping areas with these characteristics were considered high-priority for improving HEMS accessibility and are referred to as target locations. The target locations (marked by stars) are shown in Figure 5.

In Figure 5, forty target locations were identified, primarily in the northeastern and southwestern regions of CB province. Specifically, there were 26 locations in the city of Choongju (district 179), seven locations in the city of Jecheon (district 178), six locations in the County of Goesan (district 172), and one location in the County of Okcheon (district 176). Figure 5 illustrates the extent of the road network covered by the 15-min aggregated EMS response time from EMS stations (denoted by the red line) and the 10-min patient transport time from target locations (denoted by the green line). Only four target locations in Goesan County fall within the 15-min accessibility coverage.

**Figure 6:**
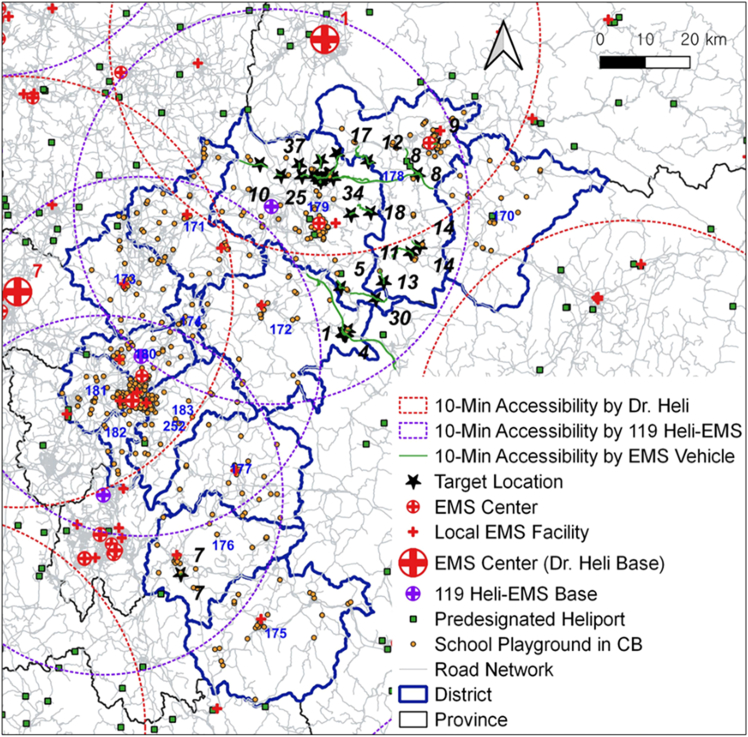
10-min accessibility by EMS vehicle or helicopter

Figure 6 illustrates both the 10-min patient transport coverage of EMS helicopters from their bases (shown as dotted rings) and that of EMS vehicles from the target locations (indicated by green lines).

In Figure 6, no existing EMS centers or local EMS facilities were identified within the 10-min EMS vehicle accessibility coverage of patient transport from any of the 40 target locations. To address this, the study examined whether existing EMS helicopters could reach all target locations within a 10-min threshold for patient transport. The 10-min accessibility coverage of HEMS is represented by a dotted ring buffer around each EMS helicopter base, as shown in Figure 6. This buffer was generated using the average cruise speed data of each EMS helicopter, which provides a more realistic measure than the maximum cruise speed data.

### Test bed-based HEMS access simulation scenario

As an initial step toward one of the main objectives of this study—developing a prototype virtual 3D visualization platform for HEMS access training—a test bed area was selected to simulate patient transport through the coordination of EMS vehicles and helicopters. Among the previously identified target locations in the northeastern districts of CB Province, seven locations had more than three crash occurrences. From these, one site with nearby HEMS infrastructure—including an EMS station, EMS center, helicopter bases, and landing sites—was selected as the test bed for the simulation. The chosen test bed location is the Sancheok Intersection in Choongju City (latitude 37.081769°, longitude 127.95459°). The nearest HEMS infrastructure includes the Mokhaeng EMS station, Konkuk University EMS center, GW Dr. Heli base in Wonju City, CB 119 Heli-EMS base in Choongju City, the Choongju Gymnasium (predesignated heliport), and the playground of Sancheok Middle School (sub-heliport).

For the daytime HEMS access simulation, the patient transport routes from the test bed to the definitive hospital were as follows: the road route connected the Mokhaeng EMS Station to the playground of Sancheok middle school via the test bed, while the aerial route involved helicopter transport from the GW Dr. Heli base to the same playground. Notably, the GW Dr. Heli base is an EMS center with a rooftop helipad.

For the nighttime simulation, the CB 119 Heli-EMS base replaced the GW Dr. Heli base due to the latter’s inability to operate at night. Accordingly, the nighttime route involved direct aerial transport from the CB 119 Heli-EMS base to the Konkuk University EMS center, with a stop at the playground of Sancheok middle school—the closest landing site to the target location—for patient transfer from the EMS vehicle. Since the Konkuk University EMS center does not have its own helipad, the nearby Choongju Gymnasium was used as the final landing site for the helicopter at night.

In the area covering the test bed and the surrounding HEMS infrastructure, the average taxi speed on the road network and the average cruise speed of EMS helicopters (as shown in Table 2) were applied to simulate patient transport by EMS vehicle and helicopter.

### GIS data based virtual space model

**Figure 7:**
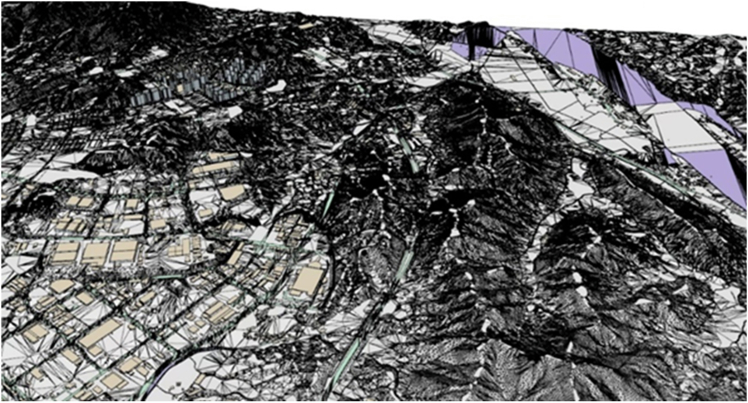
The initial 3D virtual space model generated by ArcGIS 10.8 and the SketchUP tool

The initial virtual space model created by the 3D modeling algorithm in ArcGIS 10.8 is shown in Figure 7 where four 3D layers consisting of terrain, buildings, roads, and water systems with metadata are all in vector data format and contain coordinates. The 3D layers were automatically combined within the GIS platform, and the building took the form of LOD level 1. Because design composition on a GIS platform optimized for geographical analysis has limitations, this study created an initial virtual space model through post-processing tasks, such as mesh adjustment through SketchUP, a 3D modeling program.

### Game engine based 3D visualization and user interface (UI) configuration

**Figure 8:**
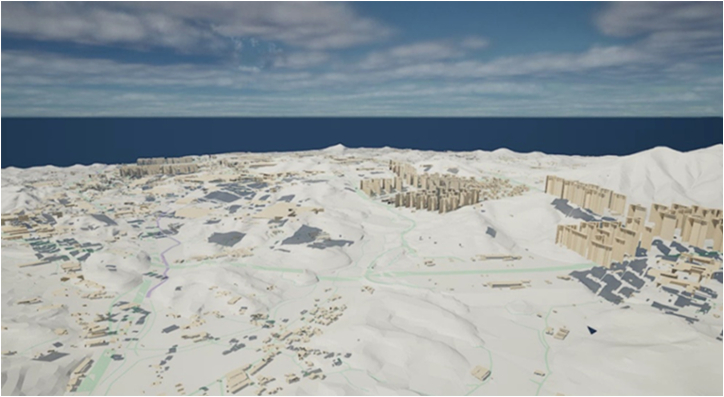
SketchUp file of test bed area imported into Unreal 5.3

Figure 8 shows the test bed area (an area in the city of Choongju) imported into Unreal 5.3 using the Datasmith plugin. The optimization of 3D model geometries imported from SketchUp was conducted with the resource inspection and remodeling around roads and patient transport paths. Next, texture mapping and lighting settings to enhance visual quality and realism were applied to the 3D resources that compose virtual environment. Additionally, essential assets including HEMS infrastructure were created for the virtual environment composition.

**Figure 9:**
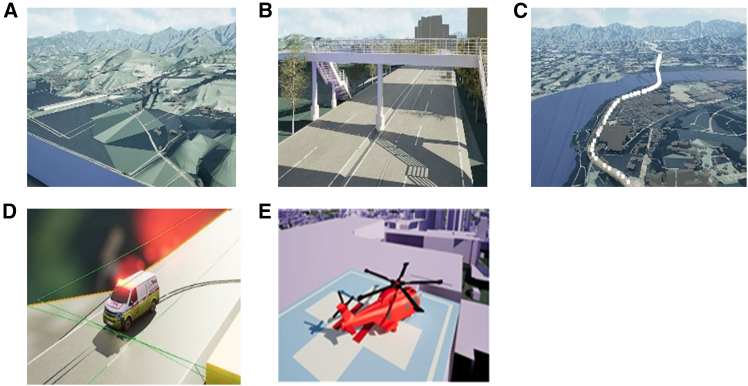
The process of building 3D virtual environment platform of HEMS access (A) Texture mapping. (B) Adjusting road environments. (C) Setting up patient transport paths. (D) Integrating with animation (EMS vehicle). (E) Integrating with animation (EMS helicopter).

After the virtual environment was completed, animations were produced by setting animation sequences for the patient transport paths by EMS vehicle and helicopter. Figure 9 shows the process of building 3D virtual environment platform of HEMS access, including texture mapping and asset creation.

**Figure 10:**
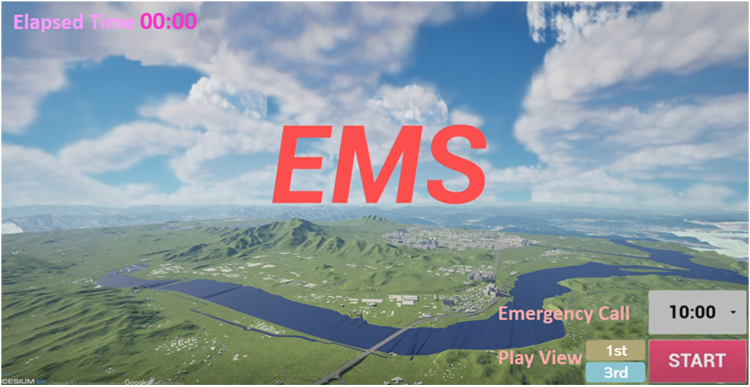
Screen shot of UI in HEMS access simulation system

The UI of HEMS access simulation system is also shown in Figure 10 where users can select the time to request patient transport and choose the viewpoint (first-person or third-person) to view the transport strategy.

In Figure 10, activating start button indicates that EMS vehicle is dispatched and EMS response begins. The proposed simulation system of HEMS access allows users (EMS vehicle and helicopter operators) to choose the time of day for patient transport between 10 a.m. and 7 p.m. In the upper left corner of Figure 10, a prehospital EMS time is also displayed, which includes: time periods traveled by EMS vehicle from EMS station to landing spot of EMS helicopter (as patient transfer point from EMS vehicle to helicopter) via the scene of crash (test bed), and time periods traveled by EMS helicopter from the patient transfer point to definitive hospital (or nearby definitive landing spot). Note that the time average taxi speed on road network of the test bed area was used to display the time periods traveled by EMS vehicle from Mokhaeng EMS station to the playground of Sancheok middle school (a patient transfer point) via the scene of fatal crash.

Also note that departure and arrival locations for EMS helicopter depend on the time of day due to the night operation issue. As provided in Table 2, the average speed of 274 km/h for AW109 helicopter at GW Dr. Heli base (a definitive hospital) was employed to display the round-trip time periods elapsed in daytime. In nighttime, the average speed of 262 km/h for H225 helicopter was employed to display the time periods elapsed from CB 119 Heli-EMS base to Choongju gymnasium predesignated heliport near Konkuk University EMS center (a definitive hospital).

**Figure 11:**
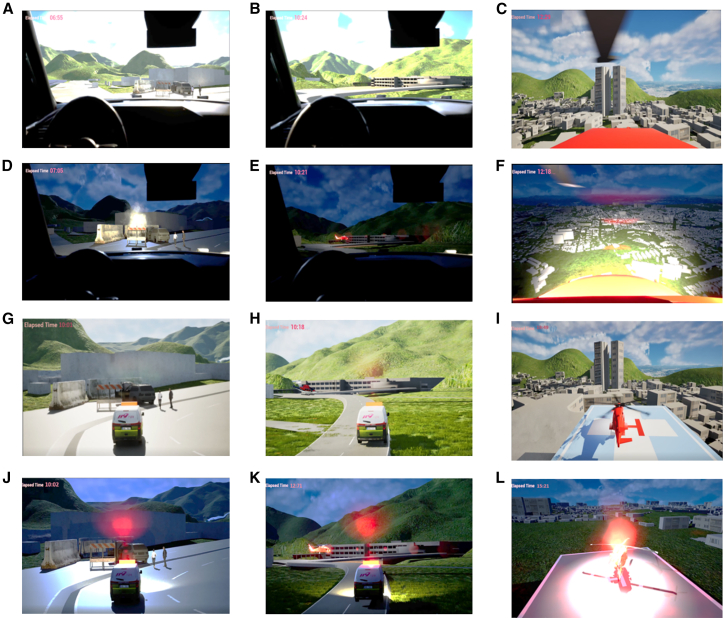
Screen shots of virtual 3D HEMS access simulation scenarios (A) Scene of crash (1st, day). (B) Patient transfer point (1st, day). (C) Definitive hospital (1st, day). (D) Scene of crash (1st, night). (E) Patient transfer point (1st, night). (F) Predesignated heliport near definitive hospital (1st, night). (G) Scene of crash (3rd, day). (H) Patient transfer point (3rd, day). (I) Definitive hospital (3rd, day). (J) Scene of crash (3rd, night). (K) Patient transfer point (3rd, night). (L) Predesignated heliport near definitive hospital (3rd, night).

The virtual platform of HEMS access displayed that the time traveled by EMS vehicle from EMS station to patient transfer point via the scene of crash is 12.8 min on the average of daytime and nighttime. Adding time periods traveled by EMS helicopter, total elapsed time for EMS vehicle and helicopter coordination based patient transport to a definitive hospital were 19.5 and 15.3 min in daytime and nighttime, respectively. A collection of screenshots by simulation scenarios in the virtual platform of HEMS access demonstrates the platform’s ability to faithfully replicate real-world HEMS access in Figure 11.

The screen shots A to C and D to F in Figure 11 show the scenarios of patient transport in the first-person view in daytime and nighttime, respectively. The screen shots G to I and J to L show the scenarios of patient transport in the third-person view in daytime and nighttime, respectively.

## Discussion

Among the 36 target locations outside the 15-min accessibility coverage shown in Figure 5, 33 are located in the cities of Choongju and Jecheon in the northern part of CB province. In particular, the city of Choongju shows a coefficient of variation in aggregated EMS response time (standard deviation divided by the average) of nearly 70%. This suggests significant disparities in response times considering factors such as the concentration of EMS stations in the city center, adverse weather conditions at the time of crashes, and challenging topography on rural principal roads. For more detailed information on the northern areas of CB province, refer to the study by Jung and Qin.^32^ The results in Figure 5 indicate that additional EMS stations could enhance coverage for the rural principal road network, especially in the northern regions of CB province.

Figure 6 shows that nearly 78% of the target locations in the northern areas of CB province fell within the 10-min HEMS accessibility coverage (denoted by the red dotted ring) of a Dr. Heli base (base no. 1). However, nine target locations in the eastern part of Goesan County (district 172), the southern parts of Choongju (district 179) and Jecheon (district 178) Cities, and the western part of Okcheon County (district 176) were outside this HEMS accessibility coverage. When considering the additional 119 Heli-EMS bases, these nine locations were included within the 10-min HEMS accessibility coverage (denoted by the purple dotted ring) provided by three nearby 119 Heli-EMS bases. This implies that utilizing existing KNFA-affiliated helicopters for EMS (119 Heli-EMS) is a cost-effective strategy for timely patient transport. EMS helicopters function as mobile EMS hospitals. When EMS helicopters are deployed, EMS vehicles must transport patients to nearby predesignated heliports within the 10-min threshold for patient transport. Among the existing predesignated heliports (denoted by green squares), only one heliport near target location No. 8 in the city of Jecheon (district 178) was within the 10-min EMS vehicle accessibility coverage (denoted by green lines).

Figure 6 also depicts all school playgrounds (denoted by yellow circles) as potential sub-heliports for future use in or around CB province. Incorporating school playgrounds as sub-heliports, in addition to predesignated heliports, allows 31 of the 40 target locations to be covered by the 10-min EMS vehicle accessibility, excluding nine target locations in the northeastern part of Choongju city (district 179). This indicates that using existing school playgrounds could be a cost-effective alternative to constructing new predesignated heliports. However, it also highlights the continued need for new predesignated heliports, especially in the northeastern part of Choongju city.

The findings indicate that, to reduce the potential for road crash fatalities, the aggregated EMS response time should be within 15 min and the patient transport time within 10 min. The results further suggest that expanding the use of 119 Heli-EMS and utilizing school playgrounds as sub-helipads or heliports can be effective strategies for timely emergency patient transport. Based on the spatial distribution of fatality-prone locations, the northeastern and western areas of CB Province should be prioritized for HEMS infrastructure expansion. In particular, additional heliports and EMS stations along principal road networks are recommended in the northeastern districts of Choongju city.

This study also developed a virtual 3D visualization platform for HEMS access training, tailored to EMS vehicle and helicopter operators. This platform was based on an assessment of road crashes and surrounding HEMS accessibility. The developed 3D virtual platform successfully visualized the complex coordination between ground and air ambulances. A notable finding from the simulation results is the discrepancy in total transport time between day (19.5 min) and night (15.3 min). Despite the lower average cruise speed of the helicopter deployed at night (H225, 262 km/h) compared to the daytime helicopter (AW109, 274 km/h), the total elapsed time was shorter at night. This is attributed to the operational strategy: the daytime scenario required a round-trip from the hospital base, whereas the nighttime scenario utilized a 119 Heli-EMS helicopter stationed closer to the crash site for a direct flight. This result quantitatively reinforces the potential efficiency of utilizing 119 Heli-EMS assets to bridge service gaps, particularly when conventional hospital-based air ambulances are unavailable or located further away.

The virtual training platform demonstrated the ability to faithfully replicate real-world HEMS access scenarios by integrating GIS data with a high-fidelity game engine (Unreal 5.3). The inclusion of diverse viewpoints serves specific training purposes. The first-person perspective provides realistic immersion for operators, simulating the actual visual field of drivers or pilots. Conversely, the third-person perspective offers broader situational awareness, allowing trainees to understand the spatial relationship between the crash location, transfer points, and surrounding terrain.

Furthermore, the simulation utilized a default 90-degree field of view (FOV) in Unreal Engine 5.3. This FOV setting is significant as it provides sufficient immersion in simulation environments, mimicking human peripheral vision to a degree that is effective for screen-based training. By validating these visual and temporal parameters, the platform suggests that game-engine-based simulations can serve as a cost-effective and flexible alternative to field training for optimizing ground-air coordination strategies.

The methods and findings of this study could guide future investments in HEMS infrastructure and provide cost-effective training for seamless coordination between ground and air ambulances. Patient transport by EMS helicopter should be prioritized in cases where an emergency occurs at a location far from the definitive care hospital, and rapid transport is essential to save the patient’s life. Accordingly, the results and tools developed in this study can effectively support decision-making aimed at identifying areas with a high probability of patient fatality, assessing the surrounding HEMS infrastructure (including ambulance stations, EMS helicopters, heliports, and hospitals), and determining the priority and spatial scope for infrastructure expansion. This study demonstrated how the proposed approach can specifically support such decisions in three aspects.

• Location planning: Leveraging random forest-based GWBL modeling, this study predicted locations with a high probability of traffic crash fatalities and found that the north eastern and western subregions of CB province should be prioritized for HEMS infrastructure expansion. Operation wise, a response time of 15 min and a transport time of 10 min (i.e., a total prehospital EMS time of 25 min) emerged as a practical and critical threshold associated with fatality reduction. Using GIS-based network analysis, this study quantified corresponding service coverage and produced ranked location choices for new sites or upgrade.
• Capacity determination: In the north eastern area of CB province where fatality-prone locations are concentrated, this study evaluated capacity expansion alternatives and showed that cost-effective measures such as utilizing existing school playgrounds as sub-heliports and, where warranted, establishing new EMS stations or heliports can close the most critical access gaps while meeting the 25-min target.
• Dispatching strategies and training: This study developed a 3D simulation-based training platform to support integrated ground-air dispatch and patient transport training within the critical prehospital EMS time threshold. This platform is a transport crew-dedicated platform that provides day/night, weather, and route variations without constraints of space, time, or cost. Accordingly, the training platform enhances crews’ route familiarity and handoff coordination, reinforcing efficient ground-air coordination strategies.

### Limitations of the study

The study acknowledges several limitations. The cost of reallocating existing HEMS infrastructure was not directly measured or compared with the cost of constructing new infrastructure. Future research could quantify the costs and trade-offs between reallocating existing HEMS infrastructure and constructing new facilities. In addition, future research will apply technologies such as LOD, texture resolution adjustment, and material integration to optimize terrain information and ensure smooth transitions between different spaces. The research will also consider diverse user interface scenarios and road environments by incorporating varying weather conditions and road features, such as pavement markings, traffic signals, and signs. Lastly, evaluations should be conducted to collect user feedback and assess performance changes using both quantitative and qualitative metrics in the future work.

## Resource availability

### Lead contact

Requests for further information and resources should be directed to and will be fulfilled by the lead contact, Sooyeon Lim (sylim@knu.ac.kr).

### Materials availability

This study did not generate unique physical materials, novel reagents, or animal models.

### Data and code availability

• Data reported in this paper will be shared in open repository (Mendeley Data).
• All custom codes can be available on request from the lead contact.
• Any additional information required to reanalyze the data reported in this paper is available from the lead contact upon request.

## Acknowledgments

This work was supported by the 10.13039/501100003725National Research Foundation of Korea (NRF) grant funded by the Korea government (10.13039/501100014188MSIT) (RS-2025-00564174).

## Author contributions

Conceptualization, data curation, funding acquisition, investigation, methodology, resources, software, formal analysis, visualization, writing— original draft, writing—review and editing, S.J. ; methodology, writing—review and editing, X.Q. ; data curation, methodology, software, visualization, writing—original draft, S.L.

## Declaration of interests

The authors declare no competing interests.

## STAR★Methods

### Key resources table

**Table:** 

REAGENT or RESOURCE	SOURCE	IDENTIFIER
**Deposited data**		

All data are available in the folder titled “DATA_accessibility analysis” within the Mendeley Data repository.	This paper	Mendeley Data: https://doi.org/10.17632/z6ty3ycyk7.2
The video titled “EMS.mp4” is available in Mendeley Data repository.	This paper	Mendeley Data: https://doi.org/10.17632/z6ty3ycyk7.2

**Software and algorithms**		

R 4.4.1	R Foundation	https://www.r-project.org/
SPSS 27	IBM	https://www.ibm.com/products
GWR 4.09	Maynooth Univ.	https://gwr.maynoothuniversity.ie/gwr4-software/
QGIS 3.22.13	GPL	https://qgis.org/
ArcGIS 10.8	ESRI	https://www.esri.com/en-us/home
Blender 4.0	Blender Foundation	https://www.blender.org/
SketchUP Pro 2023	Trimble	https://sketchup.trimble.com/ko-kr
Unreal Engine 5.3	Epic Games, Inc.	https://www.unrealengine.com/ko

### Method details

#### Road crash victim data

The current study utilized data on medical treatments for road crash victims transported to Konkuk University Hospital in CB province, from 2011 to 2016. The dataset for road crash victims included various fields, such as medical treatment outcomes, prehospital EMS time stamps, spatiotemporal conditions, collision specifics, victim’s physical information, and vehicle details. Records of instantaneous deaths at the crash scene were excluded, as these were not influenced by prehospital EMS time components. The outcome of medical treatments—whether the victim survived or died within 30 days after being transported to a definitive EMS center—was the focus of this study. Consequently, the study analyzed 568 cases, including 32 fatalities and 536 survivors. The dependent variable, the outcome of medical treatments, was categorized into fatality or survival to identify specific prehospital EMS time periods associated with each outcome.

Ground transportation based prehospital EMS time is typically divided into three periods.^6^ EMS vehicle response time (the time from when an emergency call is received until the EMS vehicle arrives at the crash scene), on-scene time (the period from when the EMS vehicle arrives at the scene until it leaves), and transport time (the duration from when the EMS vehicle departs the crash scene until it arrives at a definitive hospital). In the dataset for this study, many time-stamp records, particularly those indicating when the EMS vehicle arrived at the crash scene, were missing. Consequently, the study divided prehospital EMS time into two components: aggregated EMS response time (which combines EMS vehicle response time and on-scene time) and transport time.

#### Data preparation for EMS stations and definitive hospitals

Each fire station in Korea operates an EMS station, which runs at least one EMS vehicle unit and dispatches it to road crash scenes. According to KNFA information from 2021, there are a total of 1100 EMS stations distributed across Korea.^10^ Korea is divided into nine provinces and one island: Gyeonggi (GG), Gangwon (GW), Choongbuk (CB), Choongnam (CN), Gyeongbuk (GB), Gyeongnam (GN), Jeonbuk (JB), Jeonnam (JN), and Jeju (JJ). The Gyeonggi (GG) province, which includes the capital city, has approximately one-third of all EMS stations, the highest rate in Korea. In contrast, Choongbuk (CB), a central province, has only 4% of the total EMS stations, the lowest rate except for Jeju Island.

According to the National Emergency Medical Portal, there are 400 hospitals in Korea capable of providing emergency medical treatments. These hospitals are classified as either EMS centers or local EMS facilities.^33^ EMS centers are high-level hospitals equipped to provide advanced emergency medical care for severe crash victims, with 167 such centers existing in Korea. The remaining 233 hospitals are local EMS facilities, which offer standard emergency medical treatments. This study also considers local EMS facilities as potential definitive hospitals for severe crash victims, given that the KMHW plan includes additional designations of EMS centers.

The GG province has the highest number of EMS centers (78), while CB province has the lowest (six). Currently, eight EMS centers operate KMHW-affiliated EMS helicopters, known as Dr. Heli in Korea. Dr. Heli bases are located in seven out of the nine provinces, with CB and GN lacking Dr. Heli bases.

#### Data preparation for helicopters and heliports for HEMS

In Korea, Dr. Heli is a KMHW-affiliated helicopter used exclusively for EMS, equipped with specialized medical doctors on board. Currently, a total of nine helicopters are operated as Dr. Heli in Korea. For serious emergencies involving severe external injuries, cardiac arrest, or stroke, EMS vehicle units typically request a KMHW-affiliated air ambulance. These helicopters are dispatched to transport patients to the nearest definitive hospital. However, KMHW-affiliated helicopters are not available at night due to equipment shortages and legal restrictions. To address this, KNFA has supported one helicopter for night operations.

In 2023, KNFA initiated a HEMS project because the existing KMHW-affiliated helicopters do not sufficiently cover all areas of Korea at all times. While standard KNFA-affiliated helicopters are used for firefighting or mountain rescues, they do not have onboard medical doctors. The HEMS project aims to increase the number of KNFA-affiliated helicopters that provide medical treatments to severe emergency patients, with specialized medical doctors on board, known as 119 Heli-EMS in Korea. Currently, there are 30 KNFA-affiliated helicopters stationed at 20 bases in Korea, which could be candidates for the 119 Heli-EMS program.

In Korea, air ambulances can only land at predesignated heliports, and EMS vehicle units must transfer patients to these heliports.^5^ Currently, there are 882 predesignated heliports distributed across seven Korean provinces (GG, GW, CB, CN, GB, JB, and JN). Note that two provinces (GN and JJ Island) do not have any predesignated heliports. Longitude and latitude data for all predesignated heliports were obtained from the National Medical Center.

Due to budgetary constraints and resident complaints about helicopter noise, there are insufficient heliports for HEMS, leading to longer patient transport times.^16^ As an alternative, school playgrounds have been considered as secondary landing sites for air ambulances. For instance, the capital province of Korea recently signed a memorandum of understanding to use school playgrounds as secondary heliports in collaboration with air ambulance base hospitals. A previous study found that air medical transport time using only designated heliports was approximately five times longer than if school playgrounds were included as landing options.^16^

Consequently, this study examined existing Korean school playgrounds as potential secondary heliports for HEMS. Based on Korean regulations for the size of landing pads at predesignated heliports,^34^ this study identified 819 school playgrounds in CB province that are larger than the standard landing pad size. The dataset for each school’s name, address, and playground size was provided by the Korean Educational Statistics Service.^35^

#### RF-based key variable selection

Using R 4.4.1, this study employed an RF model to identify key variables that significantly influence road crash fatalities among the variables listed in Table 1. The RF method is effective in addressing variable multicollinearity and prioritizing the importance of variables.^36^^,^^37^ The RF algorithm works by using an ensemble of randomized classification and regression trees. In this study, the Mean Decrease in Gini (MDG) was used to measure each variable’s contribution to the homogeneity of nodes within the trees. A larger MDG value indicates a more important variable in predicting fatality occurrences. The MDG is calculated as described by Jung and Qin.^32^

(Equation 1) $$MDG_{k}\left(X_{i}\right)=1-\sum_{j=1}^{J}p^{2}\left(j|k\right)$$

where *MDG*_k_ (*X*_*i*_) = gini impurity coefficient of variable *X*_i_ at node *k*, p(*j*|*k*) = probability of class *j* at node *k*, *j* = the number of classes.

#### GWBL regression model

In this study, GWBL and comparative standard Binary Logistic (BL) regression models were developed using SPSS 27 and GWR 4.09. The GWBL model extends BL model by incorporating spatial diversity into the estimation process. Given the study’s objectives, the dependent variable is binary (fatality = 1 or survival = 0), and a major task is to identify how the local impacts of independent variables on the dependent variable vary spatially. Additionally, temporal factors were not thoroughly addressed in the RF results. Therefore, this study employed the GWBL model to fulfill the study objectives, as described by Jung et al.^36^:

(Equation 2) $$Pi\;\left(Y\right)=\exp\left\{\alpha \left(u_{i},v_{i}\right)+\beta \left(u_{i},v_{i}\right)X\right\}/\left[1+\exp\left\{\alpha \left(u_{i},v_{i}\right)+\beta \left(u_{i},v_{i}\right)X\right\}\right]$$

where *P*_*i*_ (*Y*) = probability of outcome *Y* at location *i*, (*u*_i_, *v*_i_) = two-dimensional coordinates of location *i*, *α* (*u*_i_, *v*_*i*_) = vector of constant at location *i*, *β* (*u*_*i*_,*v*_*i*_) = [*XTW*(*u*_*i*_, *v*_i_)X]^−1^*XTW*(*u*_i_,*v*_i_)*Y* = vector of local coefficients at location *i*, *X* = a set of explanatory variables, *W*(*u*_*i*_,*v*_*i*_) = weighting matrix whose diagonal elements are the geographical weights of each observation for the regression point and whose off-diagonal elements are zero.

To generate a weighting matrix in geographically weighted regression, Gaussian fixed kernels or bisquare adaptive kernels are commonly used geographic kernel types.^38^ The Gaussian kernel provides weights that continuously and gradually decrease from the center of the kernel but never reach zero.^38^ In the fixed kernel method, the geographic extent for local model fitting—used to estimate geographically local coefficients—remains constant across space, whereas the adaptive kernel allows this local extent to vary.^38^ For building a GWBL model with an unbalanced outcome distribution, the Gaussian adaptive kernel is considered a more robust option compared to the bi-square adaptive kernel.^38^

#### GIS based network analysis

The road network dataset and the locations of HEMS infrastructure were loaded into QGIS 3.22.13. The service area tool within QGIS was then used to determine the extent of the road network covered from a specific HEMS infrastructure (e.g., EMS station) based on thresholds for aggregated EMS vehicle response and transport times. During this process, the lengths of road links and average taxi speeds for each road link were utilized to calculate the road network coverage within the specified time thresholds.^39^ Using average taxi speed data provides a more accurate reflection of real-time traffic conditions compared to relying solely on posted speed limits. Additionally, the analysis accounted for two-way traffic directions.

#### GIS data based virtual space modeling

The virtual space model in this study was generated from geospatial data using GIS engine. The geospatial data were collected from the National Land Information Platform (https://map.ngii.go.kr/mn/mainPage.do) provided by Korea National Geographic Information Institute.^40^ Each layer in the GIS platform contains metadata that includes attribute information within the spatial data, allowing visualization in separated or integrated forms. Due to the extensive volume of data, essential layers including terrain, transportation, buildings, and hydrography were initially filtered. Metadata for terrain, transportation, buildings, and hydrography were collected according to major classification of terrains. The secondary data processing was conducted using ArcGIS 10.8 to minimize the volume of data, and facilitate the smooth implementation of a large-scale 3D virtual space model. Specifically, data preprocessing was carried out using ArcMap, and 3D models of terrain, transportation, buildings, and hydrography were generated through the ArcScene application according to vector and triangulated irregular network (TIN) data types in the database.

This study generated 3D models through LOD (level of detail) stages to archive benefits in terms of performance, visual quality, data management, and real-time rendering. To achieve the improvements through the LOD stages, ArcGIS 10.8 and the 3D modeling tool (SketchUp) were used in parallel by converting the WRL file format generated in ArcGIS 10.8 into a file that can be used in SketchUp. For the conversion, Blender software was employed. Features such as points, polylines, and polygons were used among four file formats collected (feature classes, shape files, coverages, and raster). For terrain models, polylines were converted into a TIN structure model. For building, road, and hydrography models, polygons were used to convert them into 3D vector models. The initial model generated by the GIS algorithm included buildings modeled at LOD stage 1, and the integration of facility models with the terrain was facilitated by the inherent location data within each dataset. While the GIS engine’s native application allows for basic graphic tasks such as color changes and boundary adjustments, its functionality is very limited. In this study, a real-time development platform of game engine (Unreal 5.3) was utilized to implement a more realistic virtual space model through the integration of external data, enhanced interactivity, and real-time rendering.

#### Game engine based 3D visualization and UI configuration

The Unreal 5.3 engine provides extensive libraries and application programming interface (API) to easily access various external data. To achieve realistic visualization and real-time interaction, this consolidated model was imported into Unreal Engine 5.3 using the Datasmith plugin. To create an immersive 3D virtual space, it is necessary to optimize imported 3D models and configure the environment. Invisible internal structures and excessively detailed components in the imported models were removed to improve performance and reduce rendering time. To optimize the performance of 3D models, LOD techniques were applied to convert them into simplified models with lower detail. After completing the 3D model optimization, tex-ture mapping was performed to enhance visual quality and realism. During texture mapping, the resolu-tion of texture files was adequately adjusted to avoid issues with excessive file size and increased ren-dering time. Subsequently, environment settings and lighting adjustments were carried out to ensure overall visual quality and fine-tune details. A specific plugin, namely sun position calculator, was also activated to implement the real-time sun position in the virtual space model of Unreal 5.3. The sun position calculator plugin allows users to dynamically simulate day and night changes by updating the sun’s position, date, and time settings.

Furthermore, a user-friendly interface for HEMS access simulation system was created. The created HEMS access simulation included several scenarios for EMS vehicle and helicopter coordination based patient transport. The UI of HEMS simulation system included a menu to select the user’s viewpoint and emergency call time. Two perspectives (first person vs. third person) were provided to experience diverse circumstances in the virtual environment. The first-person perspective maximizes the immersion of patient transport training for users (EMS vehicle or helicopter operators) because the users feel as if they are actually at the scene. The third-person perspective allows users to easily identify the location with surrounding environments and to more clearly understand patient transport route.

### Quantification and statistical analysis

In this study, the RF algorithm split the road crash victim cases into a training set (60%) and a test set (40%) to train the model and evaluate its classification performance, respectively. The RF algorithm achieved reasonable prediction accuracies for fatality (68% for the training set and 77% for the test set), survival (81% for the training set and 85% for the test set), and overall cases (78% for the training set and 84% for the test set). The RF process involved generating 500 trees and reached a minimum stable error rate of 9.3%.

The GWBL and standard BL regression models were employed to generate and compare results. The same sample size (*n* = 568) was used for both models. GWBL and standard BL modeling were conducted at a 95% confidence level (α = 0.05). For the model comparison, we utilized deviance (−2 times the log likelihood of the fitted model), the Akaike Information Criterion (AIC), and pseudo R^2^ to assess the overall model’s goodness-of-fit. Smaller deviance and AIC values, along with a greater pseudo R^2^, indicate better goodness-of-fit. Additionally, the z-value at a 95% confidence interval was used to evaluate the local coefficient estimates from the GWBL model. A 95% confidence interval suggests that an independent variable is statistically significant to the dependent variable if the maximum absolute z-value of the local coefficient estimated by the GWBL model is greater than 1.96.^41^
